# Blood parameters of wild-caught harbor seals (*Phoca vitulina*) in the German North Sea: reference intervals and potential influencing factors

**DOI:** 10.3389/fvets.2026.1898173

**Published:** 2026-09-01

**Authors:** Stephanie Gross, Andreas Ruser, Ursula Siebert

**Affiliations:** 1 Institute for Terrestrial and Aquatic Wildlife Research, University of Veterinary Medicine Hannover, Foundation, Buesum, Germany; 2 Aarhus Universitet Institut for Ecoscience Roskilde, Roskilde, Denmark

**Keywords:** blood chemistry, hematology, pinnipeds, Wadden Sea, wildlife

## Abstract

Reference ranges for blood parameters are essential for diagnostic assessment and treatment decisions for harbor seals (*Phoca vitulina*) in rehabilitation or under permanent human care. Moreover, they are also important for evaluating the health status of free-ranging animals, thereby allowing conclusions about population health. To date, robust datasets of free-ranging presumably healthy individuals are rare. Here, we evaluated reference values for 14 hematologic and 21 serum biochemical parameters of 576 harbor seals that were wild-caught in the German North Sea over almost three decades. After the removal of outliers, nonparametric or bootstrapping methods were applied, depending on the sample size for each parameter, to estimate 95% reference intervals and their 90% confidence intervals. Additionally, we investigated the influence of three factors, as well as potential time trends, on each blood parameter using the Mann–Whitney-U test. Here, the factors which showed a moderate effect on only a few of the tested blood parameters were age class, sex, and season. LDH values were higher in juveniles than in multi-year animals, while creatine showed higher values in multi-year animals. Females showed higher values for iron, BUN, and triglycerides compared to males. Red cell distribution width (RDW), total-protein and cholesterol values were higher in spring than in autumn. No specific changes over time were determined for any of the blood parameters. This study enhances the knowledge on blood reference values of free-ranging harbor seals, delivering important baseline data especially for further health assessments of the Wadden Sea harbor seal population. Continuous health examinations of free-ranging marine mammal populations represent an important early warning tool for identifying potential threats affecting these animals. Therefore, they can provide valuable information to support effective management and conservation measures.

## 1 Introduction

Harbor seals (*Phoca vitulina*) are one of the two resident seal species in the trilateral Wadden Sea (1). In the 20th century, the Wadden Sea harbor seal population suffered a strong decline specifically due to hunting (2, 3). After enforcement of national and international protection efforts the population started to recover (4) but was then affected by two Phocine distemper virus epidemics (1988/1989, 2002) and one influenza A epidemic (2014) (1, 5, 6). Although, the population has remained stable at a high level over the last decade, recent observations suggest a slight decline for reasons that are still unknown (7). Specifically, recruitment of pups into the breeding age class seems to be highly reduced, indicating a low survival rate of pups particularly in their first year (7), which highlights the need for continuous monitoring of this seal population. Furthermore, health data of marine mammal populations are often based on investigations of dead animals (8–11). While these data are extremely valuable in determining live history as well as causes of disease and death, they are subject to a sampling bias toward diseased and young animals (8–11). Here, clinical parameters of wild-caught individuals can deliver important supplemental information about adult animals (12). This is of specific importance as it allows the detection of decreasing, nonlethal health conditions which, for example, can be caused by increasing stress levels (13). In this context, health parameters of live free-ranging harbor seals can be an important early warning tool (12).

To assess health, blood parameters are widely used in human and veterinary medicine (14, 15). In addition to determining the health status of individuals, blood parameters have been successfully used to evaluate the health status of wildlife populations, including marine mammals (12, 16). However, obtaining reliable reference values representing physiologically healthy ranges in statistically robust sample sizes remains challenging (12). Additionally, several intrinsic factors have been shown to influence blood parameters, including age class, sex, season, reproductive status, geographic location, and diet (17–23). Another bias may arise when reference values are derived from animals with compromised health conditions, e.g., from animals admitted to rehabilitation facilities (24). But, although free-ranging animals are often considered apparently healthy, this might not be true for single individuals (22). Furthermore, blood parameters generally exhibit a certain degree of individual variability (25), while extrinsic factors such as sample collection and handling procedures, as well as the use of various analytical devices and statistical methods, may also influence the measured values (25, 26).

Available harbor seal blood reference intervals are of limited value for the health assessment of wild harbor seal populations, as older animals predominate in wild populations, while most published blood values were obtained from pups in rehabilitation centers (27–30), with some studies including data from older animals in rehabilitation or captive settings (21, 29). Sampled free-ranging harbor seals were also mainly pups (24, 28, 31) with limited information available for adults (26), except for one recent study on blood chemistry values of adult free-ranging harbor seals in the United Kingdom (32).

Therefore, blood reference values derived from wild, older harbor seals are essential for accurately assessing population health. Here, we investigated hematology and serum chemistry values obtained from regularly performed health monitoring captures of free-ranging harbor seals in the German Wadden Sea of Schleswig-Holstein. The unique dataset of samples from 576, juvenile and multi-year animals over almost three decades allowed a robust evaluation of population-based reference intervals for 35 blood parameters. Data analysis was performed in accordance with the American Society for Veterinary Clinical Pathology (ASVCP) 2011 consensus guidelines (25). Additionally, the current study investigates the influence of the factors season, age class and sex on the blood values. Despite being of interest for individual animals under human care, these data are important information on baseline health parameters of the wild harbor seal population of the Wadden Sea.

## 2 Methods

### 2.1 Sampling

**Figure 1:**
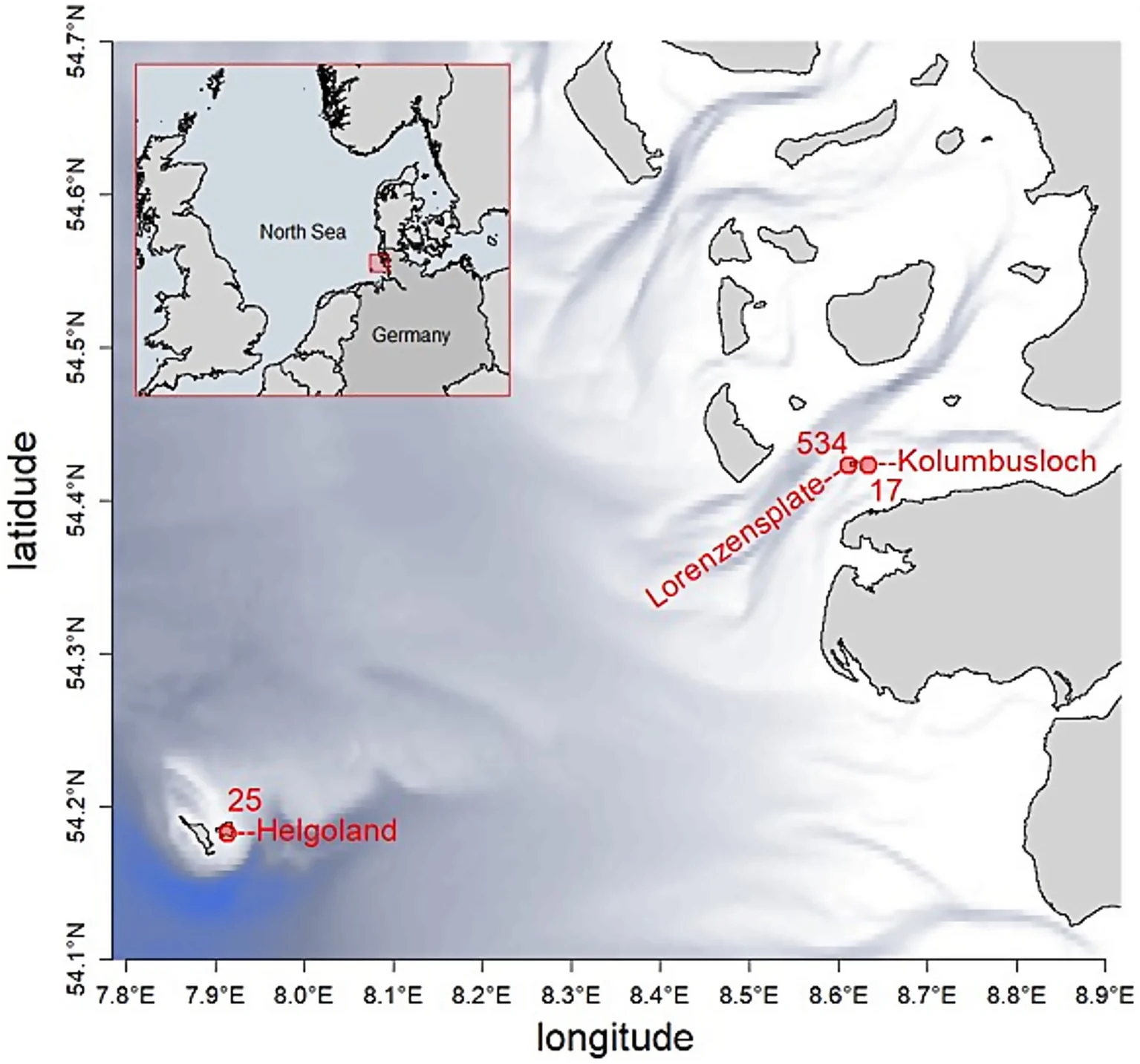
Sampling sites and numbers of sampled harbor seals per site in the German North Sea including the two sandbanks Lorenzensplate and Kolumbusloch as well as the island of Helgoland.

Free-ranging harbor seals were caught in three different locations in the German North Sea, namely on the sandbanks Lorenzensplate and Kolumbusloch as well as on the island of Helgoland (Figure 1).

Two different methods of capture were used for harbor seals. First, seals hauling out on sandbanks (Lorenzensplate and Kolumbusloch) were approached using two motorboats stretching a 100 m long and 10 m deep net between them. While fleeing into the water, seals were caught in the net, hauled onto the sandbank and individually transferred into hoop nets (33). Up to 20 animals were caught per attempt. These captures were routinely performed twice per year, once in spring and once in autumn. Second, on beaches of Helgoland, individual seals were approached by foot, caught using pole nets and sampled either directly in the pole net or after transfer into a hoop net. Following sample collection, all animals were directly released into the wild.

For most animals, sex, age class, total length and weight were determined. Sampling was performed under manual restraint without the use of anesthetics. Blood samples were collected from the epidural intravertebral vein using needles selected according to the size and condition of the individual animal. In general, 1.2 × 100 mm sized needles were used, but for smaller and/or lean animals the needle size was 0.9 × 70 mm, and for large and/or fat animals 1.1 × 120 mm. Blood was collected using syringes and immediately transferred into tubes containing ethylenediaminetetraacetic acid (EDTA) and lithium-heparin, respectively. Tubes were kept at room temperature and generally analyzed within 24 h.

Between 1997 and 2024, blood samples with a total number of 576 wild-caught harbor seals were collected and hematology as well as blood chemistry were performed. In total, 35 parameters were collected including erythrocytes (RBC) in T/L, hemoglobin (Hb) in g/dL, hematocrit (HCT) in %, mean corpuscular volume (MCV) in fL, mean corpuscular hemoglobin (MCH) in pg./Ery, mean corpuscular hemoglobin concentration (MCHC) in g/dL, red blood cell distribution width (RDW) in %, platelets in G/L, mean platelet volume (MPV) in fL, leukocytes (WBC) in G/L, granulocytes in % and 10^9^/L, lymphocytes in % and 10^9^/L, monocytes in % and 10^9^/L, eosinophiles in %, total protein in g/dl, albumin in g/dl, globulin in g/dl, serum-glucose (Glc) in mmol/L, triglycerides in mmol/L, blood urea nitrogen (BUN) in mg/dL, urea in mg/dL, creatinine in μmol/L, total bilirubin (bilirubin) in μmol/L, cholesterol in mmol/L, alkaline phosphatase (ALP) in U/L, alanine aminotransaminase (ALT) in U/L, aspartate aminotransferase (AST) in U/L, gamma-glutamyl transferase (GGT) in U/L, alpha-amylase in U/L, lactate dehydrogenase (LDH) in U/L, creatine kinase (CK) in U/L, calcium in mmol/L, serum inorganic phosphate (phosphorus) in mmol/L, sodium in mmol/L, potassium in mmol/L, and iron in μmol/L. Urea values were converted into BUN using the following equation: urea (mg/dL) × 0.467 = BUN (mg/dL) (Kraft 1999).

### 2.2 Blood analyses

From 1997 to 2001, the automated hematology analyzer Abbott Cell Dyn 3,500 (Diamond Diagnostics, Holliston, MA, USA) was used for complete blood counts from EDTA blood samples. From 2001 onwards, the device was changed to a ScilVet ABC (Scil Animal Care Company GmbH, Viernheim, Germany). In the interim phase, blood samples were analyzed in parallel with both devices providing similar results. For blood chemistry, lithium-heparin blood samples were centrifuged (15 min at 3000 rpm) to obtain plasma which was first analyzed in two different external veterinary laboratories (1997–1999 and 2000–2012) and then, from 2013 onwards, in house via a VetScan VS2 (ABAXIS Europe GmbH, Griesheim, Germany). As not all parameters were determined in some animals, the sample size per parameter is variable but indicated for each parameter in the Results section.

### 2.3 Data analyses

Data analysis was performed in accordance with Friedrichs et al. (25) who described methods for evaluating reference intervals in accordance with the ASVCP 2011 consensus guidelines. First, data for each blood parameter was plotted over time using a boxplot, histogram, and corresponding norm curve to visually identify outliers. Outliers were removed by performing data filtering using the Horn method (34). The statistics on all samples were performed in R (35) (with RStudio 2023.12.1 + 401 ‘Ocean Storm Release’) using the package ‘referenceIntervals’ (36). For the initial filtering the function ‘horn.outliers’ was used on the parameters. The lower and upper reference interval (RI) and their confidence intervals (CI) were determined using the RefLimit function, with 95% coverage for the RI and 90% for the CI. For sample sizes of 120 and higher the RefLimit function used nonparametric methods for RI and CI, while for sample sizes smaller than 120 bootstrapping methods were used for the CI. The CIs provide an estimate of the uncertainty of the limits and should generally be narrower for larger samples sizes. The CI should not exceed 0.2 times the width of the RI (25). If the width of the RI exceeded 0.2 for individual parameters, visual inspection of the distribution still showed extreme values that were not identified by the Horn method. Affected blood parameters were filtered a second time using Tukey’s method (blood parameters marked with * in Table 1).

**Table 1:** Sample size (*n*) for the different categories of the three investigated factors age-class, sex and season.

Factor	Category	*n*	Category	*n*	Category	*n*
Age-class	Juvenile	115				
			
	Multi-year	454				
			
Sex	Female	262	Juvenile	55		
			Multi-year	205		
	Male	310	Juvenile	60		
			Multi-year	249		
Season	Spring	272	Juvenile	67	Female	25
					Male	42
			Multi-year	204	Female	71
					Male	133
	Summer	66	Juvenile	5	Female	4
					Male	1
			Multi-year	61	Female	35
					Male	26
	Autumn	222	Juvenile	39	Female	23
					Male	16
			Multi-year	177	Female	96
					Male	81
	Winter	16	Juvenile	4	Female	3
					Male	1
			Multi-year	12	Female	3
					Male	9

Discrepancies in sample numbers are due to missing data (age class not specified = 7 times, sex not specified = 4 times).

Sampled animals were grouped into two age classes based on their appearance and morphometrics, while also considering that the main pupping months for harbor seals in the North Sea are June and July (37): juvenile (animals born during the sample year or the preceding year, being approximately two to 18 months), and multiyear (animals 19 months and older). Furthermore, samples were grouped, depending on the sampling date, into the four seasons “spring” (March 1 to Mai 31), “summer” (June 1 to August 31), autumn” (September 1 to November 30), and “winter” (December 1 to February 28/29). Table 2 indicates the number of individuals for both sexes, the two age classes and all four seasons.

**Table 2:** Reference intervals of blood parameters are indicated by lower and upper reference values (Low-RefVal and High-RefVal).

Blood parameter	Unit	Low-Conf	Low-RefVal	Median	High-RefVal	High-Conf	*n*	out
RBC	T/L	(3.83–4.02)	3.91	4.79	5.48	(5.40–5.54)	488	9.0%
Hb	g/dL	(15.70–16.80)	16.50	20.10	23.00	(22.80–23.40)	506	2.9%
HCT	%	(42.70–45.00)	44.11	55.35	63.60	(62.60–64.30)	510	2.3%
MCV	fL	(106.00–109.00)	108.00	116.00	123.00	(122.00–124.00)	457	11.1%
MCH	pg/Ery	(37.40–38.50)	37.90	42.30	46.60	(46.30–47.20)	477	7.2%
MCHC	g/dL	(32.20–33.00)	33.00	36.50	40.56	(40.00–40.90)	495	3.7%
RDW *	%	(13.50–13.80)	13.70	15.30	17.50	(17.40–17.70)	450	2.0%
Platelets	G/L	(96.00–128.00)	111.30	339.00	501.90	(486.00–508.00)	523	2.4%
MPV **	fL	(6.30–6.50)	6.43	7.10	8.37	(8.10–8.50)	252	3.8%
WBC	G/L	(4.90–5.80)	5.40	9.20	13.80	(13.20–14.40)	519	3.2%
Granulocytes	%	(22.00–30.00)	24.00	68.30	87.55	(86.90–90.00)	420	0.0%
Granulocytes	109/L	(3.70–4.10)	3.95	6.80	11.01	(10.50–11.80)	457	3.2%
Lymphocytes *	%	(6.80–9.00)	8.00	25.00	50.00	(47.00–52.00)	453	1.3%
Lymphocytes *	109/L	(0.40–0.60)	0.50	1.90	4.30	(4.10–4.70)	467	1.9%
Monocytes *	%	(1.00–1.00)	1.00	2.40	7.41	(7.00–8.00)	337	25.4%
Monocytes	109/L	(0.00–0.00)	0.00	0.20	0.70	(0.60–0.70)	469	1.5%
Eosinophiles	%	(2.70–4.40)	3.55	15.00	30.00	(28.00–32.00)	214	3.6%
Total protein	g/dL	(6.95–7.20)	7.10	8.20	9.80	(9.66–9.90)	495	0.8%
Albumin	g/dL	(2.90–3.20)	3.10	4.20	4.94	(4.90–5.20)	224	0.4%
Globulin *	g/dL	(2.80–3.00)	2.90	4.00	5.52	(5.40–5.90)	193	2.5%
Glc	mmol/L	(1.83–2.83)	2.37	5.77	9.44	(9.05–9.55)	507	0.4%
Triglycerides *	mmol/L	(0.49–0.55)	0.51	0.90	1.54	(1.47–1.68)	278	7.9%
BUN *	mg/dL	(27.00–28.91)	28.00	46.84	75.81	(73.34–78.27)	498	2.9%
Creatinine	μmol/L	(44.21–53.05)	53.05	88.42	135.63	(132.63–141.47)	511	0.2%
Bilirubin *	μmol/L	(2.74–3.08)	2.92	5.82	10.26	(9.92–10.26)	441	4.3%
Cholesterol *	mmol/L	(3.83–4.17)	3.98	5.79	7.67	(7.55–8.02)	308	1.6%
ALP *	U/L	(11.00–15.00)	13.55	35.00	102.00	(98.00–104.00)	461	8.3%
ALT *	U/L	(21.00–25.00)	23.00	54.00	118.70	(111.00–122.00)	465	9.4%
AST *	U/L	(31.00–42.00)	33.25	85.00	207.25	(199.00–225.00)	289	8.0%
GGT *	U/L	(5.00–6.00)	5.00	11.00	17.00	(17.00–18.00)	259	3.7%
Alpha-amylase *	U/L	(321.00–377.00)	355.45	510.00	655.00	(640.00–679.00)	202	9.4%
LDH *	U/L	(454.00–487.00)	465.75	773.00	1292.25	(1260.00–1365.00)	274	4.9%
CK **	U/L	(15.30–58.80)	44.90	319.00	1160.45	(1061.90–1411.85)	117	10.7%
Calcium	mmol/L	(2.15–2.23)	2.20	2.42	2.67	(2.64–2.69)	468	5.5%
Phosphorus	mmol/L	(0.77–0.90)	0.83	1.81	3.32	(3.15–3.51)	510	0.2%
Sodium	mmol/L	(140.00–142.00)	141.00	150.00	158.00	(157.00–159.00)	481	2.8%
Potassium *	mmol/L	(3.30–3.45)	3.40	4.17	5.20	(5.08–5.30)	465	4.5%
Iron **	μmol/L	(12.30–15.70)	13.61	26.20	41.75	(39.80–45.80)	270	4.9%

Additionally, the median and the confidence intervals of the reference values (Low-Conf and High-Conf) are shown as well as the sample size (*n*) per parameter and the percentage of removed outliers from the original sample size (out). * Blood values that were filtered a second time for outliers (Tukey’s method); ** Blood values for which CI/RI exceeds 0.2 after the second outlier filtration.

To investigate whether the selected factors have an influence, all blood parameters were tested for differences regarding the two age classes ‘juvenile’ and ‘multiyear’, the two sexes ‘male’ and ‘female’ as well as the three seasons ‘spring’, ‘summer’, and ‘autumn’. To gain insight into the multivariate structure of the data, a principal component analysis of the hematological and serum biochemical parameters was performed, followed by a permutational multivariate analysis of variance (PERMANOVA; ‘adonis2’ function in the ‘vegan’ R package) (38). Samples from ‘winter’ were excluded for further analyses due to insufficient sample size (*n* = 16). The data were checked for normality using the Shapiro–Wilk test (39). As most parameters did not meet the normality assumption, the Mann–Whitney-U test (MWU test), a non-parametric statistical test, was used to test for differences between the selected factors for all blood parameters (40). The Wilcox effect size (41) for all categories of the factors ‘age-class’, ‘sex’ and ‘season’ was tested on all parameters (package ‘rstatic’, version 0.7.2). Differences are classified as ‘none’, ‘small’, ‘moderate’ and ‘larger’.

To assess changes in blood parameter values over time, a trend analysis was conducted by grouping the data in 7-year intervals (1997–2003, 2004–2010, 2011–2017, 2018–2024). Similar to the analysis of the selected factors, the values of the parameters of the pooled years were tested against each other using the MWU test (40) and effect sized were evaluated using the Wilcox effect size (41) in R (package ‘rstatic’, version 0.7.2).

The here presented data was already partly published by Hasselmeier et al. 2008 (33) but limited to hematology parameters of the wild-caught harbor seals from the years 1997 to 2004.

## 3 Results

### 3.1 Reference intervals

Hematology and serum chemistry were measured in 576 wild-caught harbor seals from the German North Sea over a period of 28 years. Table 1 shows the reference intervals of 35 blood parameters as well as the sample size per parameter. Reference intervals are indicated by lower and upper reference values together with their corresponding confidence intervals. Additionally, median values are provided.

For all blood parameters the results for Horn and Tukey filtering, normal distribution, timeframe of sampling, as well as the statistical methods used are shown in the Supplementary Table S1.

### 3.2 Influencing factors

Sex, age class, and season were tested for their potential influence on all investigated blood parameters. In the analysis of the multivariate structure of data using PERMANOVA the interaction term between sex and season proved to be very statistically significant (Pr(>F) = 0.0061) for the hematological parameters, while statistical significance was also observed for the same term in the serum biochemical parameters (Pr(>F) = 0.0183). In Table 3 those parameters of the pairwise analysis are listed for which moderate or larger effect sizes were identified for the respective factors. Factors with no or small effect size are not included as their clinical relevance appears negligible.

**Table 3:** Determined influencing factors indicated by differences in values for blood parameters.

Blood parameter	Factor	Categories	Low-RefVal	Median	High-RefVal	*n*	Effect size
LDH * U/L	Age class	Juvenile	519.80	926.00	1376.60	67	Moderate
		Multi-year	459.20	729.00	1285.40	207	
Creatinine μmol/L	Age class	Juvenile	44.20	72.50	105.30	97	Moderate
		Multi-year	53.05	93.72	140.61	412	
Triglycerides * mmol/L	Sex	Female	0.66	0.99	1.67	104	Moderate
		Male	0.49	0.84	1.46	174	
RBC T/L	Season	Autumn	3.89	4.73	5.51	189	Moderate
		Summer	3.67	4.38	5.00	42	
RBC T/L	Season	Spring	4.21	4.85	5.48	242	Moderate
		Summer	3.67	4.38	5.00	42	
Hb g/dL	Season	Autumn	16.05	19.70	22.91	194	Moderate
		Summer	16.00	18.20	22.40	59	
Hb g/dL	Season	Spring	16.80	20.70	23.00	238	Larger
		Summer	16.00	18.20	22.40	59	
HCT %	Season	Autumn	42.67	54.70	63.60	192	Moderate
		Summer	41.43	49.50	56.86	59	
HCT %	Season	Spring	45.26	56.50	64.19	244	Larger
		Summer	41.43	49.50	56.86	59	
RDW* %	Season	Autumn	14.09	15.90	18.60	156	Moderate
		Spring	13.62	15.10	17.37	245	
RDW* %	Season	Autumn	14.09	15.90	18.60	156	Moderate
		Summer	13.38	15.20	16.95	44	
Granulocytes 10^9^/L	Season	Spring	4.17	7.20	11.38	228	Moderate
		Summer	2.94	5.75	8.85	46	
Total-protein g/dL	Season	Autumn	7.20	7.97	9.80	183	Moderate
		Spring	7.40	8.48	9.80	237	
Total-protein g/dL	Season	Spring	7.40	8.48	9.80	237	Moderate
		Summer	6.86	7.70	9.81	62	
Calcium mmol/L	Season	Spring	2.15	2.40	2.66	228	Moderate
		Summer	2.30	2.50	2.70	57	
Cholesterol ^*^ mmol/L	Season	Autumn	4.58	6.11	7.73	121	Moderate
		Spring	3.80	5.25	8.06	133	

Investigated factors are age class, sex and season tested by Mann–Whitney-U test including the Wilcox effect size. Shown are only parameters of moderate or larger effect size (0.3 < effect size). Blood values that were filtered a second time for outliers (Tukey’s method).

Only a few parameters were influenced by the tested factors. The factor age-class showed differences for LDH and creatinine, with juveniles having higher LDH values and lower creatinine values than multi-year animals. The factor sex demonstrated differences only for triglycerides, with females having higher triglycerides values than males. The factor season showed effects on eight blood parameters. Erythrocytes, hemoglobin and hematocrit values were higher in autumn and spring compared to summer. The red blood cell distribution width values were higher in autumn compared to spring and summer. Granulocyte counts were higher in spring than in summer, while total-protein values were higher in spring compared to autumn and summer. Calcium values were lower in spring than in summer, while cholesterol values showed a wider RI and lower median values in spring than in autumn.

### 3.3 Analysis over time

Several blood parameters showed variation among the four chosen time periods with moderate to larger effect sizes (Supplementary Table S2, Supplementary Figure S1). A continuous trend over time could only be observed for MCV and lymphocytes (%). MCV values showed a slight increase over the time periods, occasionally falling below or exceeding the evaluated reference intervals. Lymphocyte values showed a slight decrease over time, also undercutting and exceeding the evaluated reference intervals. Some parameters did not exhibit a continuous time trend but have different value levels in the first two time periods compared to the latter two. Globulin values were lower during 1997–2010 than in subsequent years, while Albumin showed the opposite trend. Total-protein values did not show effective variance over the chosen time periods. Sodium and BUN values were higher during 1997–2010 than in later years, while bilirubin showed the opposite pattern. Granulocytes percentages were clearly lower in the first two-time periods compared to 2011–2024, however this was not observed in the absolute granulocyte counts. Alkaline phosphatase values were lower and AST values higher during years 1997–2003 compared to the following time periods. Phosphorus values were higher in the years 1997–2003 than during 2011–2024.

Additionally, the analysis over time allowed to investigate potential influence on the blood values by the different blood analyzers and laboratories, respectively. In accordance with the years of change, hematological parameters were screened for variation between the first and second interval, showing slight differences for Hb, MCH, MCHC and granulocytes. Due to a higher variation in laboratories, biochemical parameters investigated for variances between the first, the second and the third interval. Parameters showing variance between at least one of these intervals include Glc, BUN, bilirubin, ALP, ALT, AST, GGT, LDH, CK, phosphorus, and sodium.

## 4 Discussion

This study presents robust hematology and blood chemistry reference intervals for harbor seals based on an exceptionally large dataset of 576 free-ranging individuals. These data are important for health assessments of individual harbor seals as well as for wild populations. The animals were sampled at three different locations within the German Wadden Sea over 28 years. Pooling data from the three sample locations was considered appropriate as harbor seals within the trilateral Wadden Sea are regarded as a single population (37) and all three locations are within the known habitat range of harbor seals (42, 43). Reference intervals were calculated for 35 blood parameters in accordance with the American Society of Veterinary Clinical Pathology guidelines (25). Additionally, as the factors age class, sex and season were previously reported as partitioning factors on blood values in marine mammals (19, 44), we investigated their influence on the blood parameters measured in this study, with seasonal effects showing greater variability than age class and sex. Nevertheless, the detected variance does not seem to be clinically significant. Only slight temporal trends were detected for MCV and lymphocyte counts.

Despite the large and valuable dataset on juvenile and adult free-ranging harbor seals in this study, some potential limitations need to be addressed. These include the circumstance that not all free-ranging animals are healthy. But as outliers were strictly removed, potential pathological values should be excluded from the here shown reference intervals. Additionally, the practiced physical restraint and handling without anesthesia are stressful specifically for free-ranging animals, which might have caused alterations in some of the blood parameters (45). For example, it is known that in phocids splenic contraction as stress response causes an increase of erythrocytes (46, 47). Furthermore, our capture method might have caused a bias toward compromised animals as healthy animals might be more reactive and faster, thereby more successful in avoiding getting caught in the net. On the other hand, in our experience, distance to the water as well as the status of alertness of the group are other factors influencing the likelihood of seals being captured. Additionally, changes of laboratories or blood analyzers might have influenced the values. In the analysis over time only slight differences can be seen in some of the blood parameters between the time frames in which different laboratories or blood analyzers were used. As the detected differences do not exceed other occurring inter-interval variances, it is difficult to assess which factor(s) might cause these variations.

### 4.1 Reference intervals

The reference intervals here are based on a reference population dominated by older animals (*n* = 454) compared to juveniles (*n* = 115), here defined as animals of approximately two to 18 months of age. Pups were not included in this study. The distribution between males and females is balanced, but the seasons spring (*n* = 272) and autumn (*n* = 222) are overrepresented compared to summer (*n* = 66), while only very few samples were taken in winter (*n* = 16). Nevertheless, at first, all blood results were pooled to obtain reference intervals. The reference intervals were largely consistent with reported values for harbor seals of varying age and from different locations of the Northern hemisphere (21, 26–31, 48). Detectable variance between the results presented here and those of other studies were especially seen for RDW, MPV, ALT, ALP, and phosphorus (all higher values than in the present study) as well as granulocytes, eosinophiles, and creatinine (all lower values than in the present study). No published values were found for granulocytes (%). Reasons for the detected variations may include varying health statuses (24), different age classes (18, 23), sex (17, 19), and diet (20).

In the following section, the variability of individual blood parameters between previous studies and the present study is discussed. For example, we found different values for absolute granulocyte numbers, including neutrophils, eosinophils and basophils, a subgroup of leukocytes and important immune cells in the defense of infections (49). Salazar-Casals et al. (30) determined slightly lower absolute granulocytes values (2.48–8.81) in rehabilitated harbor seal pups directly before release compared to this study (3.95–11.01). The here presented higher granulocyte values in the wild population may be associated with infections, inflammation and stress (50, 51), all of which generally can be seen, at least on a low level, in free-ranging animals (52, 53). Furthermore, Salazar-Casals et al. (30) determined higher RDW values (20.55–26.55%) compared to this study (13.7–17.5%). The red cell distribution width (RDW) indicates the degree of anisocytosis (heterogeneity of red blood cell sizes) (54). RDW may increase due to inflammation and poor nutritional status (51, 55) both of which are common in orphaned pups admitted for rehabilitation. As the blood was taken after 72–76 days in rehabilitation and directly before release, especially red cell blood parameters may still reflect the compromised health status at admittance due to the long lifespan of erythrocytes which is approximately 100–115 days in dogs (56). Another health parameter that can be affected indirectly by age is the number of eosinophiles that are immune cells which specifically defend against helminthic parasites (57). In the present study the percentage of eosinophiles were higher (3.55–30%) than those reported in a study from the Pacific on rehabilitated harbor seals of different age classes (94 pups, three juveniles, four adults) 2 weeks prior to release (0–9%) (29). The higher value in the present study could be based on an increased parasitic burden in free-ranging juveniles and adults (58–60), compared to (mainly) pups and animals that potentially got deworming during rehabilitation (24, 29). Similarly, serum inorganic phosphate values, an important element in enzymatic processes and cellular structure (61), appear to vary depending on age (pups/adults) and diet (wild/rehabilitation/permanently kept) as different studies on humans have shown that phosphorus levels vary with diet, time of the day, and age (62). Accordingly, in the present study the lower range values of serum inorganic phosphate were decreased (0.83–3.32 mmol/L) compared to other studies mainly based on pups in rehabilitation or captive settings (1.2–3.4 mmol/L) (21, 24, 28, 31). Furthermore, creatinine is used as a marker for the filtration function of the kidney but also depends on musculature, as it has been shown that creatinine values are positively correlated to muscle mass (higher in adults), and rhabdomyolysis (e.g., increased due to capture and restraint) (63, 64). Compared to other studies which were predominantly based on pups (26.5–123.5 μmol/L) (21, 24, 26, 28–31) the present study determined higher values for creatinine in mainly adult harbor seals (53.1–135.6 μmol/L).

When comparing the present study with the only other study based on mainly adult, free-ranging harbor seals (32), only a few of the blood parameters evaluated in both studies had similar reference intervals (calcium, sodium, and potassium). Slightly higher values were found in the Wadden Sea population compared to the UK population for granulocytes (absolute numbers), total protein, albumin, cholesterol, GGT (only for the higher reference range), and phosphorus. Slightly lower values were found in the Wadden Sea population for globulin (only for the lower reference range), glucose, creatinine (only for the higher reference range), and alpha-amylase. For WBC the UK population showed a wider RI than that observed in the harbor seals of the Wadden Sea. Noticeably higher values were detected in the Wadden Sea population for the enzymes ALT (23–118.7 U/L compared to 2.54–66.85 U/L) and ALP (13.55–102 U/L compared to 6.14–45.59 U/L). While ALT is an enzyme that occurs predominantly in liver cells, ALP is found primarily in the intestine, kidney, liver and bone (65). Increased values of these enzymes may therefore indicate diseases of the named organs. It is difficult to assess whether the observed variability between the two studies reflects normal physiological variation or is due to differences in disease status, geographic location, diet, populations or analytical devices and methods.

### 4.2 Influencing factors

The multivariate analysis indicated that blood parameters are interacting especially between the factors sex and season but also between age class and season. In total, 12 blood parameters showed variability correlated to one of the pairwise tested factors. Herein, most values remained within the evaluated reference intervals for the overall sample size, while some values fell below or exceeded the reference intervals. Compared with previously published reference values (21, 24, 26, 28–32), the determined variances in the present study are unlikely to be clinically relevant.

In the present study differences between the two age classes were only seen for LDH and creatinine, with juveniles having higher LDH values and lower creatinine values than multi-year animals. LDH has a wide tissue distribution and serum LDH increases, among others, with severe hepatic necrosis or inflammation, and muscle trauma (65). However, there seems to be an age-dependent effect as higher LDH values in juveniles were also reported in a study on White Sea bearded seals (*Erignathus barbatus barbatus*) (18). Consistent with these findings, studies on rats showed higher LDH tissue activity in animals during the growth phase, which decreased with age (66, 67). Interestingly, other variations seen in bearded seals were not detected in the present study, e.g., increased activity of aspartate aminotransferase (AST), alanine aminotransferase (ALT) and gamma-glutamyl transferase (GGT) in adults. Creatinine values are positively correlated to muscle mass (23), thus the lower values in juveniles might be based on their reduced muscle mass compared to adults.

While another study on harbor seals did not report any sex-specific difference in the blood values (26), in the present study sex-related differences were seen for triglycerides. Here, females showed higher triglycerides values than males. Triglycerides are the most common fats in the blood which can either be obtained through diet or synthetized by the body itself (68). Values are known to vary depending on the diet (17). Previous studies on cetaceans reported higher triglyceride levels in male orcas (*Orcinus orca*) than females (19), while another study reported higher values for female bottlenose dolphins (*Tursiops truncatus*) compared to males (69), indicating sex-related variations in triglycerides due to species specificity and prey selection.

In the present study, eight blood parameters showed seasonal variation in erythrocytes (RBC), hemoglobin (Hb), hematocrit (HCT), red blood cell distribution width (RDW), granulocytes, total-protein, calcium, and cholesterol. RBC, Hb, and HCT correlate with each other as with more red blood cells the hemoglobin content as well as the hematocrit increases (17). RBC, Hb, and HCT showed lower values in summer compared to spring and autumn. During summer, harbor seals have their annual breeding and molting season in the Wadden Sea (70), particularly the latter is accompanied with longer haul-out times and reduced foraging (71, 72). The reduced food intake during this time may negatively affect erythropoiesis (17). The increased RDW in autumn compared to spring and summer might be the regenerative response to the reduced RBC in summer (17). Furthermore, granulocyte values were lower in summer compared to spring. Granulocytes are polymorphonuclear leukocytes including neutrophils, eosinophils, and basophils (17) with neutrophils being the dominant cell type (73). As summer is the breeding and molting season for harbor seals (70) this may affect the immune status of the animals. However, other studies showed no influence of molting on the WBC in pack-ice seal species (74) and higher WBC in breeding males of southern elephant seals (*Mirounga leonina*) compared to breeding females and molting adults (23). On the contrary, in birds, specifically in molting mallards (*Anas platyrhynchos*), reduced WBC counts were noted (75). The lower total-protein values observed in summer, and autumn compared to spring might also be caused by the breeding and (post-)molting season accompanied by a reduced food intake (71, 72). The lower calcium values in spring compared to summer may indicate varying prey species, which were shown to influence blood calcium levels in harbor seals (21). This also applies to the lower median for cholesterol in spring compared to autumn (21).

In accordance with other studies (28, 76), we only minor variation was observed in a few blood parameters among the tested factors of age class, sex and season. Nevertheless, age class was expected to show greater variation as juveniles undergo fundamental physiological changes during growth and development (77).

### 4.3 Temporal trend

The 28 years of data have been grouped into 7-year intervals to investigate temporal trends. Of the 35 blood parameters investigated, only two, namely MCV and lymphocytes (%), showed a slight continuous trend over time. Additionally, several inter-interval variations for individual parameters were detected. The cause of these temporal variations is difficult to assess as various factors can play a role here. In general, stress has been shown to have influence on several blood parameters (78–81). Known stressors for marine mammals include anthropogenic activities, e.g., shipping traffic, offshore windfarm construction or tourism (82–85). Furthermore, extreme-weather events such as heatwaves as well as pollution are known stressors which can also influence blood parameters (79, 81, 86).

## 5 Conclusion

The present study provides valuable reference intervals for hematology and blood chemistry values of free-ranging harbor seals. While the evaluated reference intervals generally correlate with previously reported blood values of harbor seals, they indicate population specifics. Additionally, the study shows a higher influence on blood parameters for the factor season compared to sex and the two included age classes. These data are essential not only for the health assessment of individual animals, but specifically for supplementing information on the health status of wild harbor seal populations. Compared to health data obtained from deceased animals, clinical parameters from wild-caught animals allow additional assessment of nonlethal and chronic health conditions and further improve our understanding of the age-class of adults as well as healthy individuals. Therefore, this data represents an important early warning tool, enabling the detection of declining health conditions before increases in mortality become apparent. Furthermore, we emphasize the need for continuous monitoring of individual populations as different habitats can have an influence on blood values, for example by varying prey species or stress levels. Expanding baseline health assessments of the harbor seal population in the Wadden Sea to additional regions would further improve population health monitoring. With increasing anthropogenic activities and pressures, health monitoring of alive individuals is an important tool for species conservation and for guiding management decisions before mortalities occur.

## Data Availability

The original contributions presented in the study are included in the article/Supplementary material, further inquiries can be directed to the corresponding author.
